# Exercise Training and Cardiac Rehabilitation in Patients After Percutaneous Coronary Intervention: Comprehensive Assessment and Prescription

**DOI:** 10.3390/jcm14051607

**Published:** 2025-02-27

**Authors:** Cristina Andreea Adam, John Erskine, Buket Akinci, Tim Kambic, Edoardo Conte, Girolamo Manno, Geza Halasz, Vaida Sileikiene, Federica Fogacci, Francesco Perone

**Affiliations:** 1 Department of Medical and Surgical Specialties I, II and III, “Grigore T. Popa” University of Medicine and Pharmacy, University Street No. 16, 700115 Iași, Romania; 2 St Thomas’ Hospital, London SE1 7EH, UK; john.erskine@nhs.net; 3 Department of Physiotherapy and Rehabilitation, Faculty of Health Sciences, Biruni University, 34015 Istanbul, Turkey; barbuket@hotmail.com; 4 Biruni University Research Center (B@MER), Biruni University, 34015 Istanbul, Turkey; 5 Department of Medical Sciences in Sport, Faculty of Sport, University of Ljubljana, 1000 Ljubljana, Slovenia; tim.kambic@gmail.com; 6 Clinical Cardiology and Cardiovascular Imaging Unit, Galeazzi-Sant’Ambrogio Hospital IRCCS, 20157 Milan, Italy; edoardo.conte86@gmail.com; 7 Division of Cardiology, University Hospital Paolo Giaccone, 90127 Palermo, Italy; girolamomanno@hotmail.it; 8 Department of Health Promotion, Mother and Child Care, Internal Medicine and Medical Specialties (PROMISE) “G. D’Alessandro”, University of Palermo, 90127 Palermo, Italy; 9 Cardiology Department, Azienda Ospedaliera San Camillo Forlanini, 00152 Rome, Italy; geza.halasz@gmail.com; 10 Clinic of Cardiac and Vascular Diseases, Faculty of Medicine, Vilnius University, Ciurlionio Str. 21, 01513 Vilnius, Lithuania; sileikiene.vaida@gmail.com; 11 Hypertension and Cardiovascular Risk Research Center, Medical and Surgical Sciences Department, University of Bologna, 40138 Bologna, Italy; federicafogacci@gmail.com; 12 Cardiac Rehabilitation Unit, Rehabilitation Clinic “Villa Delle Magnolie”, Castel Morrone, 81020 Caserta, Italy

**Keywords:** cardiac rehabilitation, aerobic exercise training, resistance exercise training, percutaneous coronary intervention, acute coronary syndrome, chronic coronary syndrome, cardiovascular disease

## Abstract

Current guidelines on acute and chronic coronary syndromes recommend comprehensive and multidisciplinary exercise-based cardiac rehabilitation in Class I. Indeed, in patients after a percutaneous coronary intervention, this supervised and structured rehabilitation program improves cardiovascular risk and reduces adverse events and mortality. After an initial assessment, including a peak exercise capacity evaluation, patients follow a tailored multidisciplinary program consisting of aerobic and resistance exercise training, risk factor management, dietary counselling, physical activity counselling, weight control management, psychosocial support, and education. However, tailored management and exercise prescription require careful assessment and risk consideration of several variables such as left ventricular dysfunction, comorbidities, aging, coronary artery disease severity, physical activity capacity, and type of coronary syndrome. The functional and prognostic benefits of cardiac rehabilitation have been widely demonstrated in patients after a percutaneous coronary intervention; however, referral is still limited, although exercise should be strongly recommended to these patients in the context of cardiovascular prevention. Therefore, the aim of our article is to provide an updated, critical, and state-of-the-art review of exercise training and cardiac rehabilitation programs in patients after a percutaneous coronary intervention. Furthermore, practical approaches to the management of these patients with a multidisciplinary and personalized intervention will be provided.

## 1. Introduction

Exercise is vital in the prevention of coronary artery disease (CAD), protecting against metabolic disorders and improving quality of life [1,2,3]. Patients post revascularization of their coronary arteries should participate in exercise and cardiac rehabilitation (CR) programs [3]. Indeed, meta-analyses comparing percutaneous coronary intervention (PCI) alone with PCI and exercise have demonstrated a reduced risk of cardiac death, myocardial infarction, coronary angioplasty, angina symptoms, and re-stenosis of coronary stents [1,4,5].

Performing a percutaneous intervention can damage endothelial walls, release inflammatory cytokines, activate the clotting cascade, and poses a risk of exacerbating atherosclerosis and precipitating stent thrombosis [6]. One explanation for the benefit seen in exercise combined with PCI is the suggestion that exercise promotes the healing of damaged endothelium [1,6,7]. Exercise-based CR is a cost-effective multimodal intervention in secondary prevention and is associated with reduced cardiovascular mortality (−26%), hospitalizations (−33%) and myocardial infarction (−18%), and improved quality of life in patients with CAD [8]. Participation in exercise-based CR instead of undergoing PCI was recently shown to be cost-effective and linked to significantly fewer major adverse cardiovascular events [2]. These benefits remained unchanged even when stable CAD patients first underwent PCI and then completed exercise-based CR [9]. Despite evidence of benefit, only 20–50% of patients in the United States (US), Europe, and the United Kingdom (UK) participate in CR [7,10].

Exercise prescription in this setting is iterative using specific, measurable, achievable, relevant, and timely goals (SMARTER). Assessment and prescription should consider the frequency, intensity, time, type, and progression of training (FITT-P principle) [3,10]. Types of training include strength, aerobic, balance, or flexibility [3]. The mechanistic benefits of aerobic and resistance training in patients with heart disease are substantial. Aerobic exercise improves cardiovascular health by enhancing endothelial function, optimizing lipid profiles, regulating neurohormonal activity, reducing systemic inflammation, and increasing overall functional capacity, while resistance training complements these effects by improving muscle strength, promoting vascular homeostasis, optimizing body composition, and addressing sarcopenia and frailty [7,11]. Daily activities and exercise can be graded as low, moderate, or vigorous [12]. Current recommendations are for patients with chronic coronary syndrome (CCS) to perform in excess of 150 min per week of moderate intensity exercise (or 75 min per week of vigorous intensity exercise) [13].

It is important to commence exercise promptly post PCI as every week delaying the return to exercise requires a month of exercise to recoup the benefit [3]. Guidance, however, can vary on when it is safe to commence exercise [6], when exercise testing can occur, and the best exercise training approach [6,10,14]. Exercise prescription post PCI must be individualized to the coronaries affected, bystander disease, pre-morbid physical activity (PA), and comorbidities [3,10]. This article aims to provide healthcare professionals with an updated and critical review of exercise assessment and prescription and CR in patients after a PCI. In addition, a practical approach will be provided on how to manage these patients with a multidisciplinary and personalized intervention.

## 2. Physiology and Effects of Exercise

Physical inactivity is associated with increased risk in all-cause, cardiovascular disease (CVD) and non-CVD related deaths in patients with CAD following PCI [15,16]. Higher levels of weekly PA (expressed in metabolic equivalent task (MET)/week) is associated with greater longevity [15,16], particularly in elderly and sedentary patients with CAD with increased cardiovascular risk factors, such as smoking, diabetes, obesity, and elevated blood lipids [15,17,18]. In patients with CAD, engagement in more than 94 min/week of moderate to vigorous PA and less than four hours/day of sedentary behavior were associated with 23% and 18% reduced risk for major adverse cardiovascular events, respectively [19,20].

The best strategy to increase daily levels of PA is engagement in supervised exercise training, which presents the core component of CR programs [10,20,21]. Referral to CR of patients with stable angina elected for PCI or after the procedure is crucial, especially due to increased proportion of PCI performed in the last 23 years (+12%) [22].

Exercise training provides benefits on multiple physiological systems, including cardiac, vascular, skeletal muscle, metabolic, and inflammatory systems, and, therefore, promotes the reduction of disease burden and reduction of CVD risk factors [23,24]. Among many favorable cardiorespiratory protective mechanisms, the greatest benefit of exercise training is the increase in cardiorespiratory fitness (CRF), which is a strong predictor of all-cause mortality in patients with CAD [22,23,25]. Higher CRF is associated with a substantial reduction in all-cause mortality (−68% risk) of patients with CAD in a dose–response manner. For every 1 MET of increase in CRF, there is a 17% reduction of all-cause mortality in this patient group [26]. CRF is usually assessed using a cardiopulmonary exercise test (CPET) and is expressed as peak oxygen uptake (VO_2peak_ (mL/kg/min)). Increase in VO_2peak_ is induced by a combination of cardiac, pulmonary, and skeletal muscle adaptations following exercise training in CR [23,24]. These physiological adaptations are mainly driven by changes in cardiac morphology (increase in left-ventricular mass, compliance, and end-diastolic volume) that increase convective O_2_ transport and delivery to working muscle by increasing cardiac output and stroke volume [3]. Exercise training also stimulates greater nitric oxide production and availability, thereby providing greater vasodilatation of the arteries and expansion of blood volume for a greater transport of O_2_ to exercising skeletal muscles. In skeletal muscles, convective O_2_ transport is enhanced with increased capillarity density, capillary-to-fiber ratio, changes from fast to slow (type 1) muscle fibers, and improved mitochondrial content and function [23]. These favorable cardiac and skeletal muscles adaptations also promote changes in glucose metabolism (increased uptake in the working skeletal muscle and thereby reduction in fasting glucose and glycated hemoglobin A1c levels), blood lipids (decreased low-density lipoprotein and increased high-density lipoprotein), inflammation (reduction in C-reactive protein and interleukin-6 levels), blood pressure (reduction in systolic blood pressure via reduction in sympathetic activity), body composition (reduction in fat mass and maintenance or increase in muscle mass), psychological health (reduction in stress and symptoms of depression) [23].

Most of the favorable effects of supervised exercise training can be achieved by participating in aerobic training [27,28]; however, the combination of aerobic training with resistance training has recently been shown to enhance the benefits on VO_2peak_, maximal muscle strength, quality of life, and body composition over aerobic training alone in patients with CAD [28,29,30]. Additionally, the use of higher intensity of aerobic training [31,32] and resistance training [24,30,33,34,35] may be considered to further enhance the benefits of multimodal exercise-based CR.

## 3. Cardiac Rehabilitation in Patients After a PCI—Benefits and Barriers

Early cardiovascular recovery, started from the period of hospitalization for the acute cardiovascular episode, contributes to the improvement of morbidity–mortality, functional limitations, and quality of life, with prognostic and therapeutic implications alike. Thus, CR is an essential pillar of cardiovascular prevention in this category of patients [12]. As recommended by both the acute coronary syndromes (ACSs) and CCSs Guidelines of the European Society of Cardiology (ESC), CR is recommended for all patients after an acute coronary event, both those re-vascularized (interventional or surgical) and those treated with medication [13,36,37]. Promising results of CR after a PCI have been reported for almost 25 years, irrespective of the generation of stent used, with adherence to a personalized exercise program being associated since 2001 with a reduction in the rate of recurrence of acute cardiovascular events over the next 3 years.

The referral of patients with ACS and CCS to specialized, multidisciplinary centers for the implementation of such CR programs is supported by data from the literature that attest to the reduction of the risk of recurrence of potentially fatal ischemic events. Although the symptomatic and functional benefits (increase in walking distance, cardiac and respiratory dynamics) are proven, the referral of patients after a PCI to CR is still limited. A follow-up of a cohort of 2986 patients who underwent a CR program after a PCI showed a 33% reduction in the risk of death 6 years after the acute event. The results obtained by the same group of investigators were also dependent on the duration of the recovery program, with a 50% lower risk in patients who had completed a minimum of 36 sessions of physical training (*p* < 0.001) [38]. A multicenter study conducted in 28 centers in Norway [39] showed that patients with PCI and an acute cardiovascular event have a higher enrollment rate compared to that of patients with stable angina pectoris (3.2 times higher participation). Additionally, with respect to demographic characteristics, male gender and older age were predictors associated with non-participation in cardiovascular rehabilitation programs (*p* < 0.001), while high educational level was an argument in favor of such programs. Gender differences in referral to CR programs have been evidenced in several clinical trials published so far in the literature, all studies showing a lower referral (relative risk of 0.89 compared to that of men) and a tendency to discontinue the program (relative risk of 0.7) [23,24,25,26,27,28,29,30,31,32,33,34,35,36,37,38,39,40,41,42,43]. Smith et al. analyzed the main outcomes of CR programs according to gender and found a decrease in functional benefit at the end of the program for women secondary to a lower rate of completion of physical training, control of risk factors (especially weight management, dyslipidemia, and diabetes mellitus) and, as a result, of a poorer psychosocial balance compared to that of men [44]. In a cross-sectional clinical study that included a total of 2163 patients from 16 countries evenly distributed globally and that included 916 women (42%), different barriers associated in part by gender according to whether or not they participated in a CR program were highlighted. Thus, women who did not follow a CR program listed as main barriers the fact that they were not aware of the existence of such programs, that they were not guided towards them, the associated cost, or the tiring or painful training sessions. Among women who were enrolled in the CR program, low adherence was secondary to long distance between home and the center, poor transportation, or family responsibilities [45].

In 2007, the level of referral of patients to the CR after an acute myocardial infarction was only 13.9% [46]. More recent data report an increase in addressability of up to 40%, still insufficient by analyzing the associated risks and benefits [47]. The main identified limitations are financial (lack of cost coverage by the health systems or high co-payment), the small number of centers or them being located far away from home, and the working hours that prevent participation in the CR. A meta-analysis published in 2024 which included a total of 16 studies and a total of 1810 patients who started the CR program no earlier than the day after the PCI and no later than one month after the PCI revealed that the timing of initiation and duration of the CR program does not correlate with the occurrence of arrhythmias, intracoronary restenosis, or angina in this timeframe, but emphasized the importance of starting CR as soon as possible after discharge after a coronary event [48]. Ma et al. [49] analyzed a cohort of 473 patients with ST-segment elevation myocardial infarction (STEMI) undergoing PCI who were divided into two groups according to the completion of the CR program (104 patients who completed physical training and 369 who discontinued CR). A six-month analysis revealed an improvement in serum NT-proBNP levels (*p* = 0.027) and an increase in functional capacity assessed by a 6 min walk distance (6MWD) (*p* < 0.001) in the first group of patients compared to those in the second group. Other highlighted benefits were the decrease in acute cardiovascular events by improving endothelial dysfunction and rheological parameters (despite a higher percentage of dyslipidemic patients), with lower mean age and improvement in left ventricular ejection fraction (LVEF) (*p* < 0.001). The main factors associated with ischemic recurrence in patients who followed the CR program were being over 65 years old, smoking history, distance covered at 6 min walking test (6MWT), or LVEF [50]. A retrospective analysis has demonstrated the beneficial effect of CR programs in patients with STEMI treated interventionally with reduced pre-procedural TIMI flow [51]. Early enrollment in a CR program has been associated with reduced incidence of coronary restenosis as well as with limited left ventricular remodeling (which is directly proportional to the duration and timing of the onset of exercise training) [5,52]. Furthermore, CR is supported by the improvement of systolic function parameters in patients with STEMI and PCI, as demonstrated by Wang et al., who, analyzing a group of 180 patients, showed that CR led to a decrease in left ventricular diastolic diameter and an improvement in LVEF (*p* < 0.05) [53]. The improvement in functional, imaging, and biological parameters does not depend on the type of exercise performed, with positive evidence in both aerobic and resistance training [54,55].

**Figure 1:**
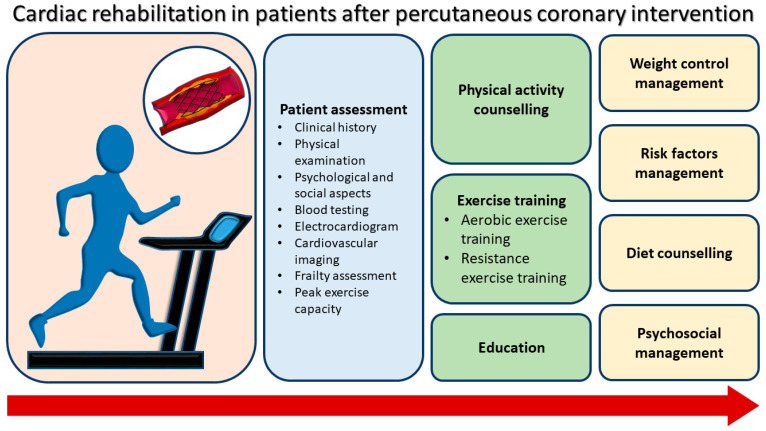
Core components of cardiac rehabilitation in patients after a percutaneous coronary intervention.

**Figure 2:**
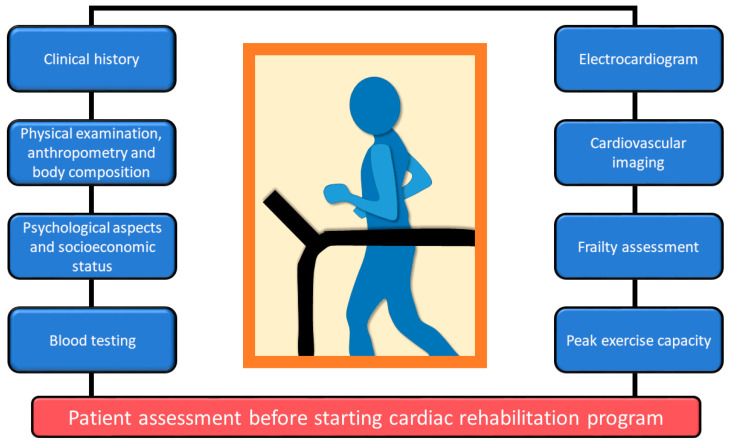
Initial evaluation of patients after a percutaneous coronary intervention before starting cardiac rehabilitation.

Core components of CR in patients after a PCI include initial assessment, PA counselling, exercise training, education, weight control management, risk factor management, diet counselling, and psychosocial management (Figure 1). During patient assessment before starting CR, peak exercise capacity is evaluated (Figure 2). All patients referred to specialized centers in order to initiate the rehabilitation program perform a CPET 1–2 weeks after stenting, the maximum limited by symptoms. The positive stress test is suggestive for the existence of a remaining ischemia in the revascularized territory or in other territories [56,57]. The first ventilatory threshold is important for determining the level of exercise intensity (the boundary between slight and moderate intensity) and is normally in the range of 50–60% of maximum oxygen consumption or 60–70% of maximum HR. In asymptomatic patients, with complete revascularization and high exercise capacity, long-term adherence to the CR program is inferior compared to lifestyle change measures and adherence to medication to improve secondary prevention outcomes, which is why it is recommended to alternate home-based CR with an institutionalized program for 1–3 weeks, three times a week [58].

Exercise and CR prescription could be suggested based on left ventricle function and functional capacity. Specifically, patients with reduced LVEF ≤ 40% or with an exercise capacity of less than 3–4 METs will follow recovery programs of prolonged duration, similar to those with heart failure [10]. It is recommended that in-center programs consist of a minimum of five training sessions per week, whereas outpatient programs are advised to include at least three training sessions per week to ensure optimal outcomes. The duration of the training will initially be 10–20 min, to which a warm-up period of 5–10 min and a recovery period of similar duration are added. Depending on the patients’ symptoms and tolerance, training will be continuous or in intervals. In deconditioned patients, interval training is preferred to improve cardiovascular fitness, endothelial function, and metabolic health while reducing the risk of overexertion through controlled rest intervals. The shorter, alternating activity–recovery structure enhances exercise tolerance and minimizes perceived exertion, increase adherence, and make it particularly effective for patients with low exercise capacity [59]. Instead, in patients with an exercise capacity of 4–5 METs, classical exercise training is recommended, and the exercise program is most often continuous, with a duration of 30–40 min (plus warm-up and cool-down) [60,61].

## 4. Cardiac Rehabilitation Program: Aerobic and Resistance Exercise Training

### 4.1. Aerobic Exercise Training

CR patients after a PCI should be stratified to low-, moderate-, and high-risk groups, and a rehabilitation program should be prescribed based on this risk [13]. Left ventricular systolic function, CAD severity and revascularization, comorbidities, and ageing influence the risk of the patient post PCI [62,63]. Exercise prescription and exercise tolerance also vary depending on ACS vs. elective PCI, bystander coronary disease, and level of activity prior to PCI [10,64]. The American Association of Cardiovascular and Pulmonary Rehabilitation risk stratification tool predicts adverse and clinical events [65]. Patients should not participate in standard CR with features of unstable angina, new ischemic changes on resting ECG, orthostatic blood pressure drop > 10 mmHg with symptoms, critical aortic stenosis, acute systemic illness, fever, or uncontrolled atrial/ventricular arrhythmias. A working group consensus developed an “everyday practice and rehabilitative training (EXPERT)” interactive tool, enabling healthcare professionals to individualize exercise prescription and therapeutic outcomes [66,67].

Exercise training should not start within 2 days post myocardial infarction. After elective PCI with radial access and femoral access, it can start the next day and week, respectively [67]. If access vessels are repaired surgically, exercise is postponed until the vessels are healed [7]. Forty-eight hours post index infarct, patients should begin walking 2–4 times per day for periods of 3–5 min at an intensity heart rate (HR) 0–20 beats above standing HR. Patients progress once 10–15 min of continuous walking is tolerated. Features indicating discontinuation of exercise include diastolic blood pressure above 110 mmHg, ventricular/atrial arrhythmias, second- or third-degree heart block, marked dyspnea, or angina. Exercise capacity evaluation can occur 4–6 days after myocardial infarction while an inpatient. Graded exercise testing is considered safe 14 to 21 days after a myocardial infarction. The ESC guidelines support exercise testing 1–2 weeks post infarct and 1 day post elective PCI [10]. Additionally, one study suggests that exercise testing can be safely performed 1 week after primary PCI [14]. The choice of testing modality should be guided by the patient’s LVEF and pre-morbid PA levels [10]. Hansen et al. emphasize the safety of exercise testing, reporting no adverse events during 277,721 patient-hours of exercise training when testing was conducted beforehand, compared to only two adverse events during 105,375 patient-hours without prior testing [67]. CPET is the gold standard to assess the peak exercise capacity; however, if the CR center does not have this method, a cycle ergometry test is suggested [24]. However, in patients who are unable to exercise on a treadmill or bicycle, 6MWT or an incremental shuttle walk test could be performed [10]. Aerobic training should be performed at least three times per week, ideally 6–7 days per week [10]. For intensity training, moderate or moderate-to-high intensity is recommended in these patients. Moderate intensity is considered 40–69 VO_2peak_ (%), 55–74 HR max (%), 40–69% HRR (%), or 12–13 on the Borg scale but varies depending on the patient’s premorbid conditioning [3]. The exercises include walking, cycling, or circuit training lasting initially for 20 min per session [10]. For patients with lower-extremity disorders or orthopedic comorbidities, stationary cycling, swimming, and water aerobics may be appropriate [7]. Progression occurs once a duration target is achieved. However, high-intensity training could be prescribed in selected patients post-PCI, for example, to increase functional capacity [3,10]. An 8-week randomized trial compared high-intensity interval training (HIIT) with moderate-intensity steady-state training (MISS). HIIT involved short bursts at >85% HRmax, while MISS consisted of steady exercise at 40–70% HRR. VO_2peak_ improved significantly more in the HIIT group at 8 weeks, but no differences were observed at 12 months. Adverse events were rare (three in HIIT, one in MISS), and withdrawal rates were similar (24 vs. 15). The findings suggest that HIIT is beneficial for certain patients, and finding no difference at 12 months suggests short-term benefits if the program is not continued at that intensity. Interestingly, attrition rates in standard CR and HITT training are similar. However, in the absence of clear evidence, moderate-intensity continuous training is suggested as the most feasible and cost-effective program [13]. After the initial and comprehensive assessment, this program is prescribed in patients at low risk. Instead, in individuals at moderate-to-high risk, aerobic exercise should start at 40% of the HRR [10]. During the program, patients should have symptom education and HR and blood pressure measurement during exercise [10,67].

HIIT has become an increasingly popular alternative to moderate intensity training in recent years, with the results of meta-analyses comparing the two training modalities supporting its safety, tolerance, lift tolerance, and effectiveness in increasing long-term adherence [68,69,70]. Based on animal models, it has been shown that intermittent ischemia induced by HIIT stimulates the development of collateral circulation without associated myocardial injury, also contributing to the improvement of endothelial dysfunction [52].

In patients with PCI, patients who underwent an HIIT-based CR program had a lower rate of re-stenosis, an effect explained by the improvement of nitric oxide-mediated endothelial vasodilation, which increases nitric oxide levels in coronary endothelial cells and thereby inhibits intimal proliferation processes [52,71]. In a study by D’Andrea et al. [72] a group of 75 patients with a recent acute coronary event was randomized into two subgroups that underwent either HIIT or MCT-based CR. The patients who followed HIIT had additional improvements in left ventricular diastolic function (*p* < 0.01), left ventricular systolic function (assessed by both ejection fraction and left ventricular global longitudinal strain, *p* < 0.01), or left atrial strain. Regarding myocardial work, it improved in the HIIT group (*p* < 0.01) and correlated most closely with VO_2peak_ assessed by CPET. Comparing HIIT and MCT, the former is associated with an additional improvement in CRF and vascular function in the short term (first 8 weeks of CR), but in the long term (after 1 year), the benefits are similar. The choice of one of the two methods also depends on the level of fitness (MCT being safer and more suitable for patients with very low physical fitness) or left ventricular systolic function (HIIT has been shown to be safe in patients with mild systolic dysfunction, while in other categories of patients, the results are limited) [73].

A meta-analysis that compared HIIT with MCT included a total of 22 clinical trials enrolling a total of 949 patients [74] has demonstrated the superiority of HIIT over MCT in improving CRF under similar safety conditions. Among patients who completed HIIT, the most significant improvements in VO_2peak_ were seen in those who completed a minimum of three HIIT sessions per week for at least 12 weeks.

Regarding exercise training, a warm-up and cool-down are suggested. Warming up lowers the ischemic threshold by increasing coronary endothelial relaxation and increasing coronary artery blood flow. A warm-up should last 10 min, with full effects suggested at 15 min, but deconditioned patients may achieve less duration. A warm-up involves large muscle groups and joint mobilization through stretching and lower-intensity aerobic work. Instead, a cool-down prevents post-exercise ischemia, arrhythmias, and hypotension. Its duration is similar to that of the warm-up and it uses exercises requiring less effort than those in the training session do and includes a 15 min observation period.

### 4.2. Resistance Training

General recommendations for PA include a combination of regular aerobic and resistance exercise throughout the week, which is also the basis of recommendations for patients after ACS [12,36]. Resistance training in addition to aerobic exercise is associated with a lower risk of total cardiovascular events and all-cause mortality in CCSs [13]. Resistance training may improve PA, cardiac function, and quality of life in patients after a PCI [55].

Patients undergoing elective PCI can begin resistance training as soon as the puncture site has healed sufficiently. This may be as early as one day after a radial artery procedure or approximately one week after a femoral artery puncture under supervision.

Exercise intensity for resistance training is commonly determined using the concept of one repetition maximum (1RM), which refers to the heaviest weight an individual can lift for a single repetition over a full range of motion [75]. Resistance training below 20% of 1RM is aerobic, while 30–50% of 1RM (15–30 repetitions) builds endurance, and 50–70% of 1RM (8–15 repetitions) optimizes strength by creating hypoxic conditions for adaptation [3].

The suggested prescription for resistance training is 1–3 sets of 8–12 repetitions at an intensity of 60–80% of 1RM for a frequency of at least two non-consecutive days per week, progressing to three days, using a variety of 8–10 different exercises involving 8–10 major muscles of the upper and lower extremities [10,13,55]. The intensity of resistance training for the upper extremities may be lower than that for the lower extremities. The resistance training prescription based on the consensus statements [3,55,75] for patients after a PCI is shown in Table 1. In higher-risk patients, the intensity of the exercise should be set at 30–40% RM [55]. High-load resistance training (70–80% of 1 RM, with 3–4 sets of 9–11 repetitions per set) may be suitable for selected well-conditioned patients after a PCI [34,75]. The Borg scale is a useful tool for monitoring perceived intensity during resistance training sessions. It can help guide the selection of a resistance level that corresponds to an initial perceived exertion rating of 11–14, indicating an effort ranging from “fairly easy” to “somewhat hard” [3,76]. Similarly, the OMNI-RES scale is an effective alternative for resistance training in older adults, with an initial intensity setting of 4–6, representing a range from “somewhat easy” to “somewhat hard” [24,77].

Dynamic resistance training is preferred to isometric resistance training, which can cause blood pressure fluctuations. Dynamic resistance training can be performed using exercise bands, free weights, or machines, with variable contractions (concentric for muscle shortening, eccentric for lengthening) and speeds. This mimics daily muscle loading, with advanced applications incorporating rapid concentric and eccentric actions at high loads. In resistance training, each repetition should last 4 s, with a 1 s concentric phase followed by a 3 s eccentric phase [78]. The rest periods between sets should be at least 60 s. It is suggested that fast lifting, with 1 s concentric and 1 s eccentric contractions, be paired with longer rest periods between sets (90 s or more) to achieve optimal results [34,75].

To enable further improvements in muscle strength, progressive resistance training is promoted in CR, where repetitions, intensity, and rest periods can be adjusted over time [3]. The progression of resistance training involves moving from basic to more advanced, functional movements. Basic training begins with generic, single-joint exercises performed in a stable, controlled environment, such as lying or sitting with slow, uni-planar movements. As training progresses, exercises become more specific and include multi-joint, multi-planar movements performed in standing positions at faster speeds and on unstable surfaces. Additional complexity may include visual deprivation, acyclic or alternating movements, and dual tasking to improve functional fitness [24,79].

Resistance exercises should be performed rhythmically at a moderate pace through a full range of motion, avoiding a tight grip, and should be stopped immediately if symptoms such as dizziness, arrhythmia, dyspnea, or angina occur [75].

Resistance training is an essential component of exercise programs for older patients, providing unique benefits beyond those of aerobic exercise. It helps prevent or reverse sarcopenia and also improves metabolic, vascular, cognitive, and mental health and frailty [80]. Incorporating flexibility, balance, and coordination training into exercise programs for older adults offers significant benefits. These modalities not only improve overall physical fitness but also play a critical role in preventing falls, which is a major concern in this population [3,55]. By improving postural stability and movement efficiency, such training promotes greater confidence and independence, thereby encouraging sustained adherence to long-term exercise programs in older post-PCI patients.

### 4.3. Breathing Exercises and Respiratory Muscle Training

In the early post-PCI phase (the first two weeks), patients should be encouraged to engage in breathing exercises in addition to gentle stretching and progressive mobilization [75]. These activities are critical in minimizing the risk of kinesiophobia and facilitating the transition to more advanced stages of exercise training. Early implementation of such interventions promotes physical and psychological readiness, ultimately supporting long-term rehabilitation outcomes.

Preliminary evidence indicates that reduced inspiratory muscle strength is presented in patients with stable angina and acute MI [81]. Similarly, patients with acute MI following revascularization also show reduced inspiratory muscle strength compared to that of controls (83–89 cmH_2_O (78–85% predicted) vs. 109 cmH_2_O (108% predicted)) [82,83]. It is known that inspiratory muscle weakness contributes to abnormal ventilatory responses during exercise and exertional dyspnea [77,78]. Therefore, inspiratory muscle training (IMT) can be considered to improve respiratory muscle performance and improve ventilatory responses during exercise, particularly in those with inspiratory muscle weakness (maximal inspiratory pressure (PI max) < 70%) after a PCI [68,84]. Suggested IMT intensity is 30% of PI max at baseline and can progress to a maximum of 60%, with intensity adjustments every 7–10 days using either threshold or resistive loading equipment. Each session should be 20–30 min per day, performed 3–5 times per week for at least eight weeks. Combining IMT with aerobic exercise or aerobic/resistance training has also been recommended [68].

Alternative techniques, including slow breathing exercises [85] and sitting baduanjin [86], have been shown to be effective in promoting recovery and offer potential benefits for patients undergoing PCI.

## 5. Tailored, Multidisciplinary, and Risk Factors Management in Clinical Practice

Essentially, CR is a multidisciplinary, integrative concept that aims to correct modifiable risk factors and regular exercise to increase exercise capacity and increase the anginal threshold in patients with residual ischemic lesions and thus increase the quality of life [87,88]. Adopting a Mediterranean diet (with the macronutrient intake adapted to the particularities of each patient—diabetic or not, with or without diabetes mellitus, with or without liver damage, with or without dyslipidemia) rich in fruits, vegetables, whole grains, heart-healthy fats, fish, and seafood helps both to correct the lipid profile and to lose weight. According to some recent data from the literature, starting from the Mediterranean diet, the concept of Mediterranean lifestyle has come to include various aspects related to food preparation, socializing, and adopting an active lifestyle [89,90].

It is known that diabetic patients suffering from acute myocardial infarction have a less favorable prognosis compared to that of patients without diabetes mellitus [91,92]. A group of investigators comparatively analyzed a group of 370 diabetic and 962 non-diabetic patients with CCS who underwent a CR program consisting of 36 outpatient exercise sessions associated with general lifestyle change measures and specific drug therapy [93]. The group of investigators demonstrated that at the onset of CR, diabetic patients had lower METs (*p* < 0.001). Physical training sessions were associated with improved exercise capacity in both groups, but the benefit was greater in diabetic patients (mean increase in METs by 1.7 vs. 2.6, *p* < 0.001). In a similar clinical study, Khorshid et al. [94] analyzed a cohort of 50 patients with acute myocardial infarction who were enrolled in a CR program 30 days after a PCI and, after a symptom-limited CPET, found a beneficial effect of exercise on HR, HR reserve, as well as baseline HR recovery in the first minute and second minutes into recovery (*p* < 0.005 for all parameters). The rate of participation in CR programs after ACS is low irrespective of the presence of diabetes mellitus, but recent data from the literature emphasize a greater decline among diabetic patients as well as reduced adherence frequently associated with early discontinuation of CR [95]. McKeever et al. [96] demonstrated a statistically significant reduction in fasting blood glucose in patients with PCI who underwent 12–24 sessions of exercise training. These results confirm the beneficial effect of participating in a CR program of at least 3 months compared to that of undergoing only one session of exercise training through a reduction of mortality of 47%, according to a Medicare study that included a total of approximately 30,000 patients.

CR programs lead to optimization of cardiovascular risk in PCI patients, including improvement/normalization of the blood pressure profile. In a clinical trial including 378 patients with CAD, 41% of the patients who completed at least 42 sessions had a systolic blood pressure reduction of at least 5 mmHg (higher in male patients). A dynamic follow-up also showed a reduction in ischemic recurrences among hypertensive and smoking patients at the beginning of the CR [97]. Prior to the widespread use of stenting, CR was the main modality for secondary and tertiary prevention in combination with drug therapy and was associated with a 30% reduction in all-cause mortality compared with drug therapy alone [98]. More precise data have been provided by Goel et al. who analyzed a cohort of over 2000 patients in an observational study and observed a 45–47% reduction in all-cause mortality in PCI patients who participated in CR, independent of gender, age, or mode of percutaneous intervention (elective or emergency) [99]. Furthermore, Song et al. [100] demonstrated among patients with acute myocardial infarction and PCI who were referred to a CR center that in patients with at least the stenosing vessels, the risk of recurrence of an acute ischemic event in the first year was lower compared to that in those who did not participate in CR. Similar results have been reported by the group of investigators also in patients with at least two stents implanted when compared to those with only one stent.

The beneficial effect in coronary and dyslipidemic patients has been demonstrated in large clinical trials, being one of the most consistent results obtained in patients who complete CR programs [101]. The reduction in serum low-density lipoprotein cholesterol (LDL-C), serum triglyceride, and total cholesterol levels (*p* < 0.001 for all parameters) was evidenced in a meta-analysis conducted by Wu et al. [102], being further evidence in support of limiting the progression of atherosclerotic processes, the occurrence of new potentially fatal acute coronary events, and the control of modifiable risk factors [8]. CR also helps achieve the ESC-recommended LDL-C targets among patients with dyslipidemia, with participation in a 3-month CR program increasing the percentage of patients with LDL below 70 mg/dL from 57% to 63% in a study of 1015 patients [103].

**Figure 3:**
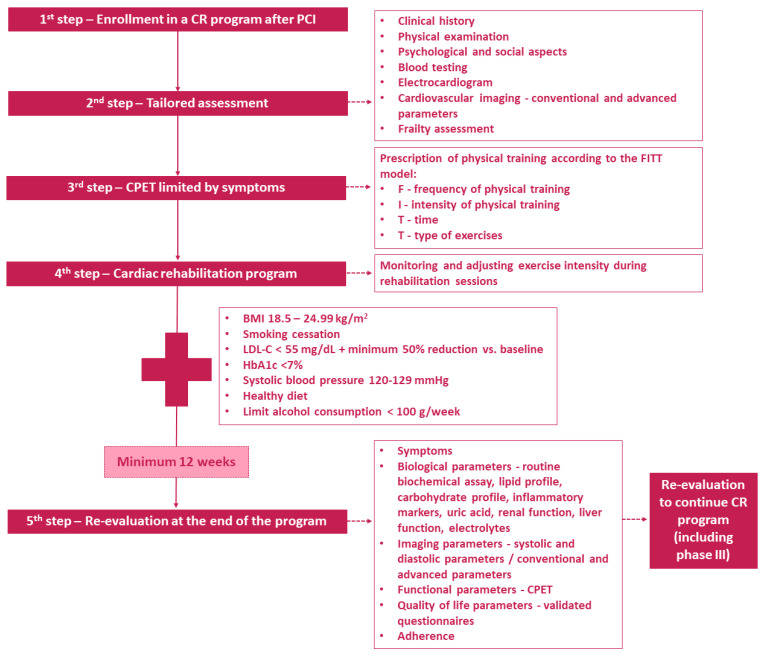
Cardiac rehabilitation program in patients after a percutaneous coronary intervention: tailored assessment and management. BMI, body mass index; CPET, cardiopulmonary exercise test; CR, cardiac rehabilitation; HbA1c, glycated hemoglobin; LDL-C, low-density lipoprotein cholesterol; PCI, percutaneous coronary intervention.

The majority of patients with coronary atherosclerotic lesions associate multiple cardiovascular risk factors with a cumulative effect on morbidity–mortality and prognosis. Diabetes mellitus, hypertension, and dyslipidemia are associated with obesity in a significant proportion of patients. Accelerated industrialization and digitalization have accentuated sedentary lifestyles and thus increased adiposity. CR contributes to the reduction of body mass index (BMI) with implicit cascade improvement of the blood pressure profile, LVEF, or parameters of lipid or carbohydrate metabolism (*p* < 0.005 for all data). Patients with CCS and a BMI ≥ 30 kg/m^2^ were associated with lower mean METs than patients with BMI values below this limit (7.97 ± 2.4 vs. 9.74 ± 2.47, *p* = 0.007) in a prospective study which included 120 patients who completed a 12-week phase 2 CR program [104]. When considering the impact of obesity on the effectiveness of CR programs, we must also consider the obesity paradox. There is ample evidence in the literature that despite similar angiographic success rates, patients with normal and very lean BMI have a higher risk of in-hospital complications, including death (*p* < 0.001) [105]. Additionally, anatomical factors such as anterior chest wall conformation, particularly pectus excavatum (PE) significantly affects cardiorespiratory function, primarily impairing right ventricular mechanics more than left ventricular mechanics [106]. It was found that PE is characterized by increased HR at the anaerobic threshold and reduced stroke volume at both the anaerobic threshold and peak exertion [107]. As the deformity worsens, cardiac output declines, reflecting true physiological impairment. Reduced exercise capacity in PE individuals stems from compromised cardiovascular performance rather than ventilatory limitations or deconditioning [108]. Together, these factors highlight the need for a nuanced approach to risk stratification based on tailored assessment and management in CR programs. Thus, it improves cardiovascular risk factors, functional capacity, and prognosis. Careful exercise monitoring and continuous re-assessment during and at the end of the program are necessary, with the goal of continuing CR in the long-term. A practical and detailed approach is suggested in Figure 3. In recent years, the concept of digital health has been implemented more and more widely in the field of CR, thus supporting a percentage of patients with low adherence to traditional CR programs in specialized centers where distance, working hours, or high cost may be barriers to low addressability [109,110,111,112]. Data in the literature indicate that home-based CR programs or hybrid approaches have the same long-term functional benefit in patients following a CR program, with similar clinical outcomes at 3–12 months [109]. Home-based CR is a feasible option for stable, low- or moderate-risk PCI patients who may choose to undergo the CR program at home with assisted monitoring via telemedicine [17].

## 6. Conclusions

CR is an essential pillar in the management of patients with PCI, having both additional therapeutic and prognostic value. Regardless of how it is performed, elective or emergency, all patients after a PCI should be referred to specialized CR centers with the aim of limiting functional decline, increasing the anginal threshold, correcting modifiable risk factors, and decreasing the risk of recurrence or occurrence of an acute cardiovascular event. The multidisciplinary and integrative approach to patients after a PCI helps to achieve an improvement in imaging and functional parameters after a personalized program of exercise and CR.

## Figures and Tables

**Table 1:** Resistance training program after a percutaneous coronary intervention.

Time After PCI	Resistance Training Program	Issues to Be Considered
Early phase (up to 2 weeks)	<30% of 1 RM or Borg ≤ 11 or OMNI-RES Scale ≤ 4 5–10 repetitions per muscle group (1–3 sets, 1–2 min rest between each set), 2 days/week	All dynamic resistance exercises should be performed with proper breathing that involves exhaling during the exertion phase and inhaling during the return or relaxation phase.
Progression phase (2–12 weeks)	30–60% 1 RM or Borg 12–13 or OMNI-RES scale 4–5 10–15 repetitions (1–3 sets, 1–2 min rest between each set), 2–3 days/week. For selected patients who can tolerate this program without any symptoms, it can progress to the following prescription: 60–80% 1 RM or Borg 13–14 or OMNI-RES Scale 5–6 8–10 repetitions (1–3 sets, 1–2 min rest between each set), 2–3 days/week.	In patients with higher risks, the intensity of the exercise should be set 30–40% of 1 RM. High-load resistance training (70–80% of 1 RM, with 3–4 sets of 9–11 repetitions per set) may also be suitable for selected well-conditioned patients.
Maintenance phase (12–24 weeks)	The resistance training program can progress as after the previous completed progression phase by incorporating position variations, transitioning to multi-joint and multi-planar movements, and integrating dual-task skills.	Engaging in resistance exercises involving complex positions may pose risks for elderly patients. Consequently, it is essential to incorporate balance and coordination exercises into their training programs to enhance safety and overall physically fitness.

PCI, percutaneous coronary intervention; RM, repetition maximum.
